# Parkinson’s disease following COVID-19: causal link or chance occurrence?

**DOI:** 10.1186/s12967-020-02670-9

**Published:** 2020-12-29

**Authors:** Wei-Shan Li, Ling-Ling Chan, Yin-Xia Chao, Eng-King Tan

**Affiliations:** National Neuroscience Institute, Duke NUS Medical School, Outram road, Singapore, 169608 Singapore

There is increasing evidence of neurological involvement in central and peripheral nervous systems in COVID-19 patients [1]. However, the association of specific neurologic syndromes with COVID-19 in some instances is unclear. Here, we reviewed published reports of “Parkinson’s disease” (PD) following COVID-19 infections and discuss the potential links and challenges. We identified 3 independent reports linking PD with COVID-19 [2–4] (from pubmed search of terms “Parkinson’s disease” and “COVID-19”) as summarized in Table1.

**Table 1:** Published Cases linking Parkinson’s disease with COVID-19

Authors	Cohen ME et al. [2]	Faber I et al. [3]	Méndez-Guerrero et al. [4]
Time of publication	Sep-2020	Aug-2020	Jul-2020
	Case 1	Case 2	Case 3
Age	45 years old	35 years old	58 years old
Gender	Male	Female	Male
Family history of parkinsonism	Nil	Nil	Nil
Use of drug that can cause parkinsonism	Nil	NIL	Nil
Length of hospital stay for initial COVID-19 infection	3 days	No hospitalization required	> 47 days
Severity of COVID-19 infection	Mild	Mild	Severe (mechanical ventilation required)
Interval between COVID-19 infection and parkinsonism	20 to 30 days	10 days	32 days
Prodromal parkinsonian symptoms	Nil	Nil	Nil
Parkinsonian features at presentation	Micrographia, hypophonia, hypomimia, tremor, cogwheel rigidity, bradykinesia	Hypomimia, eyelid retraction, slow and hypometric saccades, asymmetric bradykinesia and cogwheel rigidity, stooped posture and parkinsonian gait	Hypomimia, bradykinesia, cogwheel rigidity, postural and rest tremor
Other neurological signs	Nil	Nil	Myoclonus, decreased consciousness, opsoclonus
Cognition	Normal	Normal	Not evaluated
Response to antiparkinson drugs	Good (pramipexole and biperiden)	Good (levodopa/ benserazide)	Poor (trial of apomorphine)
Follow-up period	1 month	–	–
Clinical outcome	Improvement observed	Significant improvement observed	Spontaneous significant improvement observed
Routine blood tests	Unremarkable	Unremarkable	Unremarkable at the time when parkinsonism developed (including copper and ceruloplasmin)
CSF studies	6 white blood cells, normal glucose and protein No evidence of infection	0 cell, normal glucose and protein No evidence of infection	8 cell, normal glucose and mildly elevated protein 0.82 g/L No evidence of infection
Common neuronal antibodies	Negative in serum and CSF	–	Negative in serum and CSF
Anti-SARS-CoV-2 IgG	Detected in serum but not in CSF	–	–
RT-PCR of the CSF for SARS-CoV-2	Negative	–	Negative
MRI brain	Normal	Normal (including nigrosome-1 and neuromelanin imaging)	Normal
DaT scan	–	Decreased dopamine transporter density on the left putamen	A bilateral decrease in presynaptic dopamine uptake asymmetrically involving both putamina worse on the left side
PET scan	Decreased^18^F-FDOPA uptake in both putamen and mild decreased uptake in the left caudate	FDG-PET normal	–
MIBG	–	–	No cardiac autonomic denervation
Genetic testing	Negative	–	–

All 3 case reports revealed a temporal relationship between acute COVID-19 infection and new-onset parkinsonism with intervals ranging from 10 to 32 days. None of the patients had a family history of parkinsonism, was exposed to drugs that may cause parkinsonism or reported any prodromal parkinsonian symptoms. Except for the patient reported by Méndez-Guerrero et al. [4] being critically ill and requiring mechanical ventilation, the other 2 patients had only relatively mild COVID-19 infection. There was no evidence of brain COVID-19 infection (from spinal tap) nor were the MRI brain scans abnormal, although positron emission tomography (PET) scan and dopamine transporter (DaT) scan did reveal asymmetric findings in all 3 cases. While clinical improvement was observed in all the cases, only 2 patients responded clinically to dopamine agonist or levodopa.

The authors of these reports have suggested a possible causal relationship between COVID-19 infection and new-onset parkinsonism. However, we would like to draw attention to some caveats that have not received attention.

First, Cohen ME et al. [2] in their report did not specifically state if a thorough neurological examination has excluded signs of parkinsonism at the time of admission for COVID-19 infection. Since viral parkinsonism is far less common than PD [5], it is possible that these reported patients may already have preexisting PD but the symptoms/signs were unmasked by the acute viral infection.

Second, there was no neuroimaging evidence of inflammation or structural damage in basal ganglia or olfactory tract, nor evidence of COVID-19 infection in the cerebral spinal fluid (CSF). Neuroimaging involvement of thalamus, basal ganglia and substantia nigra as well as positive CSF viral markers are common findings seen in viral parkinsonism [10]. Given the normal MRI brain scans and unremarkable CSF studies in all the cases, alternative causes other than the acute COVID-19 infection should be entertained.

Interestingly, these reports [2–4] have not excluded the possibility of a chance occurrence of COVID-19 infection in patients who have undiagnosed PD. However, the asymmetric findings on PET scan or DaT scan seen in all 3 cases and a fairly good response to dopamine agonist or levodopa in cases reported by Cohen et al. and Faber et al. [2, 3] are in keeping with PD. Furthermore, any form of sepsis frequently exacerbates parkinsonian symptoms, especially hand tremor. The suggested association will be much stronger if there was no clinical evidence of parkinsonism on detailed examination upon admission and longer term follow up information is available to show a complete recovery or a static course of the symptoms.

Viral parkinsonism or postencephalitic parkinsonism, though rare, has been well recognized for decades and can be caused by a variety of viruses [6]. Encephalitis lethargica, for instance, was deemed to have a very close etiologic relationship with postencephalitic parkinsonism [7]though some have argued that the etiology of postencephalitic parkinsonism may be more complex and multifactorial [8]. The exact mechanism leading to the presumed degeneration of nigrostriatal dopaminergic neurons after a viral infection is still unclear. Proposed hypotheses include virus-induced inflammation contributing to neurodegeneration [9], and “multiple hit” damage [10].

In summary, while the reports of PD following COVID-19 infection are intriguing, more concrete data are needed to support their causal link and the purported direct invasion of COVID-19 into the basal ganglia in the absence of any clinical or neuroimaging evidence of encephalitis.

## Data Availability

Not applicable.
